# Thyroid Hormones in Conventional and Organic Farmers in Thailand

**DOI:** 10.3390/ijerph16152704

**Published:** 2019-07-29

**Authors:** Pornpimol Kongtip, Noppanun Nankongnab, Nichcha Kallayanatham, Ritthirong Pundee, Nattagorn Choochouy, Jutharak Yimsabai, Susan Woskie

**Affiliations:** 1 Department of Occupational Health and Safety, Faculty of Public Health, Mahidol University, 420/1 Rajvidhi Road, Bangkok 10400, Thailand; 2 Center of Excellence on Environmental Health and Toxicology, EHT, Bangkok 10400, Thailand; 3 Mahidol University, Nakhonsawan Campus, Nakhon Sawan 60000, Thailand; 4 Faculty of Public Health, Thammasat University Lampang Campus, Lampang 52190, Thailand; 5 Buddhachinaraj Phitsanulok, 90 Sithamma traipidok Road, Muang, Phitsanulok 65000, Thailand; 6 Department of Public Health, University of Massachusetts Lowell, One University Ave, Lowell, MA 01854-2867, USA

**Keywords:** conventional farmers, organic farmers, pesticides, thyroid hormone

## Abstract

Pesticides can act as endocrine disruptors by different mechanisms including inhibition of iodine absorption, increases in thyroid hormone clearance, decreased cellular uptake of thyroid hormones, or changes in expression of thyroid hormone regulated genes. This study examined how exposure to pesticides impacts thyroid hormone levels, thyroid stimulating hormone (TSH), triiodothyronine (T3), thyroxine (T4), free T3 (FT3), and free T4 (FT4) by comparing conventional (*n* = 195) and organic farmers (*n* = 222), and by evaluating which types of pesticides might be associated with changes in thyroid hormone levels. Questionnaires were used to collect information about farmer characteristics, self-reported stress, agricultural activities, and history of pesticide use. Conventional farmers were asked to report the type and quantity of pesticides used each day. The TSH, FT3, T3, and T4 levels of conventional farmers were 1.6, 1.2, 1.3, and 1.1 times higher than those of organic farmers, respectively, after adjusting for covariates. Several specific herbicides had a significant relationship between the amount applied and an increase in thyroid hormone levels, after covariate adjustment. They included: paraquat (TSH, FT3 and T3); acetochlor (FT4); atrazine (TSH, FT3 and T3); glyphosate (T4); diuron (TSH) and the “other” herbicides including alachlor, propanil, and butachlor (FT4 and T3). The most commonly used herbicide among conventional farmers was glyphosate, followed by paraquat, 2,4-dichlorophenoxyacetic acid (2,4-D). These findings suggest that exposure to pesticides could impact the development of metabolic diseases and other health outcomes by altering the endocrine system (the thyroid hormone levels) through the hypothalamic–pituitary–thyroid (HPT) axis. This work is a part of a longitudinal study which will evaluate the sub-chronic effects of repeated exposure to different types of pesticides on thyroid hormone levels.

## 1. Introduction

Agricultural pesticide use is increasing in the Southeast Asia. Pesticides were first imported into Thailand in 1966 [1], and import levels have continued to rise since then. The highest volume of pesticides imported are herbicides (75.1%), followed by insecticides (10.9%), fungicides (10.0%), and other pesticides (3.9%) [2]. Currently, Thailand is facing a challenging problem of over the use of pesticides, posing potential increased health risk to agricultural workers, consumers, and to the environment [1,3]. In response, the Thai government has developed and supported several programs including integrated pest management (IPM), good agricultural practices (GAP), and organic agriculture [4]. Organic agriculture (no use of synthetic pesticides or fertilizers) became part of King Rama IX’s Sufficiency Economy programs. The Royal projects were started in the early 1970s to improve the living standards of hill tribe people and to promote new farming methods in the northern provinces. The Alternative Agriculture Network (AAN) was set up around 1984 to foster sustainable agriculture activities in Thailand [5]. However, organic farming in Thailand remains in an early stage; the main products are rice, vegetables, and fruit. The reasons conventional farmers give for changing to organic farming include greater profits due to the higher prices for organic produce, better health for themselves and their families, and better soil fertility [5].

Some pesticides have been reported to act as endocrine disruptors with effects on many systems, including the thyroid [6,7]. In human epidemiological studies, significant changes of serum lipid levels occur as a result of thyroid dysfunction, and a significant reduction in PON1 activity (polymorphism that impacts metabolism of pesticides and other toxic chemicals) was observed in both hyperthyroid and hypothyroid patients [8]. Among Mexican floriculture workers, increasing exposure to organophosphate pesticides was associated with increased TSH and T4 levels and decreased T3 levels, after accounting for PON1 activity [9]. A cross-season study of serum samples from greenhouse workers exposed to pesticides showed that free T4 and free T3 and T3 levels were reduced, and TSH levels were increased from the start to the end of the spraying season [10]. In rats, glyphosate disrupts the hypothalamic-pituitary-thyroid (HPT) axis, with pesticide exposed groups showing decreased TSH concentrations, but no variation in the levels of T3 and T4 between the exposed and control groups [11]. In a small study of pyrethroid manufacturing workers in Egypt, exposed workers had significantly lower levels of T3 and T4, but higher TSH compared to unexposed university administrative workers [12]. In a cross-sectional study of farm residents in Brazil, the total lifetime years of exposure to fungicides, herbicides, and dithiocarbamates was associated with increased TSH and decreased in FT4 among men [13]. Other widely used pesticides such as acetochlor and cyhalothrin have been tested in zebrafish and amphibian models and have been shown to affect the development and metamorphosis that could affect HPT axis and thyroid function [14,15,16]. However, further studies in humans are necessary to clarify the impact of pesticide use in agriculture on thyroid hormone levels. The aim of this cross-sectional study was to compare thyroid hormone levels (TSH, T3, T4, FT3 and FT4) between organic and conventional pesticide using farmers, and to examine whether particular types of pesticides could be identified as impacting thyroid hormone levels.

## 2. Materials and Methods

### 2.1. Study Population

This cross-sectional study used health promoting hospitals, public health volunteers, provincial public health officers, and community leaders to recruit conventional farmers, rice farmers, and vegetable farmers in two sub-districts of Phitsanulok Province, and sugarcane farmers in one sub-district of Nakhon Sawan Province, as well as organic farmers in five sub-districts in Yasothon Province. The organic farmers grew rice, fruit, and vegetables. Inclusion criteria included males or female farmers over 18 years old who were free of a current self-reported diagnosis of diabetes, high blood pressure, or thyroid or heart disease. For conventional farmers, one farmer who sprayed pesticides was selected from each household; in general, these were most likely to be male. Organic farmers had to use certified organic practices for all of their crops. Where possible, we recruited male organic farmers to represent the household; however, more often females were the active field workers. This study was approved by the Ethical Review Committee for Human Research, Faculty of Public Health, Mahidol University, (MUPH 2015-146).

### 2.2. Data Collection

The subjects were recruited in February–April 2016 and a baseline physical health examination was conducted in November and December of the same year. The questionnaires comprised farmer personal health characteristics, self-reported stress, home and demographic information, self-reported health problems, agricultural activities, and history of pesticide use. For conventional farmers, every time they sprayed pesticides, they had to fill in a diary that included information about the type of pesticide sprayed, quantity of pesticide used, area sprayed, and any health symptoms after spraying. The farmers were trained to fill in the diary at the health center, and at the beginning, the site officer also went to their home to help them fill in the diary and train other family members to help. The site officer contacted the farmer every two weeks to check on their progress, and diaries were collected regularly throughout the year. The daily moles of pesticide used were calculated by identifying the volume of pesticide applied each day and multiplying by the density of the product, the fraction of active ingredient in the product, and the molar mass of the active ingredient. The annual amount of pesticide used was calculated by summing all the moles of active ingredient applied across the year and for each type of pesticide (insecticide, fungicide and herbicide), summing the annual moles of each pesticide type (Equation below).

$$\begin{array}{l} Cumulative\;moles\;pesticide\\ =\overset{n}{\underset{day\;1}{\sum }}\left(ml\;product\;applied\times density\;\frac{g}{ml}\times fraction\;active\;ingredient\times \frac{moles}{g}\;active\;ingredient\right) \end{array}$$

Data on weight, height, waist circumference, body composition (Tanita model DC-360, Amsterdam, The Netherland), blood pressure (taken twice 10 min apart and averaged), and blood samples were collected at 7–9 am in the morning. Sera extracted from blood samples in non-heparinized vacutainer tubes were stored at −20 °C until analysis. All samples were analyzed at the Buddhachinaraj Hospital, the regional medical center in Phitsanulok province, using standard clinical laboratory methods. They included: serum glucose, triglycerides (TG), total cholesterol (TC), high density lipoprotein (HDL), low density lipoprotein (LDL) measured by AU5800 (Beckmann Coulter, Atlanta, GA, USA) [17,18], and the analysis of TSH, T3, T4, FT3, and FT4, a paramagnetic particle used as a chemiluminescent immunoassay for the quantitative determination of human TSH, FT3, FT4, T3, and T4 levels in human serum using the Access Immunoassay Systems with UniCel DxI 800 Access Immunoassay System, Beckman Coulter (Atlanta, Georgia, USA) [19,20,21,22,23]. The limit of detection was 0.005 μIU/mL for TSH, 0.09 ng/dL for FT3, 0.15 ng/dL for FT4, 0.01 μg/dL for T3 and 0.5 μg/dL for T4. For BMI, we measured weight (kg) and height (cm); the BMI = weight (kg)/(height, m)^2^.

### 2.3. Statistical Analysis

Descriptive analysis of the demographic characteristics and clinical outcomes (thyroid hormone, cholesterol and triglyceride level) was done using Chi Square, Fisher Exact test and independent *t* tests using SPSS (version18; PASW Statistics Base 18, Serial no. 5082368, ID no. 5071846) from SPSS (Thailand) Co., Ltd., Khet Huai Khwang, Thailand. Comparison of thyroid hormone levels between conventional farmers and organic farmers used a general linear model with a natural log of the thyroid hormone levels (TSH, T3, T4, FT3, FT4) as the outcome, due to the skewed distribution of thyroid levels. Univariate models examined other potential covariates such as sex, age, current smoking, current drinking, insecticide use at home, stress, eating fruits and vegetables less than half a kilogram 5–7 days/week, and dichotomous variables based on clinical normal vs abnormal levels including body fat%, BMI, waist circumference, triglyceride, cholesterol, HDL, LDL, and metabolic syndrome. When parameters were associated significantly with thyroid levels in the univariate models, they were included in the multivariable linear model (sex, current smoking, current drinking alcohol, insecticide use at home in the past year, triglyceride levels, and stress in the past 2–4 weeks). The moles of active pesticide ingredient sprayed each day by the farmer were calculated from the diary entries of the conventional farmers and summed for a year. For categories of pesticides (insecticide, herbicide, fungicide), the moles of individual pesticides applied were summed over the year. Since organic farmers did not use any chemical pesticides, 0 mole of active ingredient was assigned for each category of pesticides for them.

## 3. Results

The average age of organic farmers (53.1 years) was significantly higher than that of the conventional farmers (50.0 years) (Table 1).

More conventional farmers were male (73.8%) than organic farmers (54.1%), their education and marital status were also significantly different, as was their current alcohol use, current smoking, and insecticide use at home in the past year. The prevalence of abnormal total cholesterol, HDL, LDL, and BMI were also significantly different between the two groups. Their working hours in any agricultural activities and the percentage with current second jobs were not significantly different. Stress symptoms in the past 2–4 weeks, including difficulty sleeping, reduced concentration, irritation, boredom, or not wanting to meet people were significantly different between the two groups.

### 3.1. Comparison between Conventional Farmers and Organic Farmers

The percentage of abnormal clinical levels was significantly higher among organic farmers for FT3, T3, and T4 but not for TSH, which is primarily used to diagnosis thyroid disease. These abnormal levels were below the clinical norm and represent a hypothyroid status (Table 1). A simple comparison between the means of the natural log of the thyroid hormone levels for conventional farmers versus organic farmers found significant differences for TSH, FT3, and T3, with levels for these hormones being higher among chemical farmers (Table 2).

Multivariable linear regression models for the natural log of TSH, FT3, T3, and T4 were adjusted for other covariates (sex, current smoking, current alcohol use, insecticide use at home in the past year, triglyceride levels, and any stress symptoms in the past 2–4 weeks). Since these are log linear models, the parameter estimates were exponentiated so that they became multipliers, showing that the hormone levels of conventional farmers were significantly higher, i.e., by a factor of 1.61 for TSH, 1.16 for FT3, 1.34 for T3, and 1.13 for T4, after controlling for all covariates (Table 3).

When the categorical variable for conventional vs organic farmer was replaced with the number of moles of the active ingredient sprayed during the year for different categories of pesticides (insecticide, herbicide, fungicide), the adjusted linear regression models showed that there was a small but significant increase in TSH for each mole of herbicide sprayed (Table 4).

### 3.2. Effect of Herbicide Exposure to Thyroid Hormone

Conventional farmers use a variety of herbicides (Table 5). Glyphosate was most commonly used (69.2%), followed by paraquat, 2,4-D, acetochlor, “other” herbicides, diuron, amethrin, and atrazine. The category of “other” herbicides also included alachlor, propanil, and butachlor. It should be noted that most farmers use a combination of herbicides, fungicides, and insecticides; many reported using multiple herbicides. Amethrin was the herbicide applied in the highest concentration (moles of active ingredient), followed by diuron, acetochlor, atrazine, 2,4-D, paraquat, glyphosate, and other herbicides (Table 5).

When individual linear regression models for the natural log of TSH, FT3, T3, and T4 included the number of moles of the specific herbicide sprayed during the year, each mole of paraquat sprayed was found to significantly increase TSH, FT3, and T3, while spraying more moles of acetochlor increased FT4 (Table 6).

Each mole of atrazine sprayed was found to increase TSH and T3 and decrease FT4. Increasing moles of glyphosate sprayed was found to increase T4, and spraying more moles of diuron resulted in increased levels of TSH, while spraying more of the herbicides in the “other” category, which included alachlor, propanil, and butachlor, was found to increase FT3 and T3 (Table 6).

## 4. Discussion 

Previous studies have reported that certain insecticides, herbicides, and fungicides are endocrine or thyroid disruptors [24,25,26]. This study found that the simple geometric mean levels of TSH, FT3, and T3 for conventional (pesticide using) farmers were significantly higher than those organic farmers. However, there could be many possible explanations for this difference. We found that the age and education of organic farmers were significantly higher than those of conventional farmers, probably because organic farmers were originally conventional farmers. Other authors have noted that being older and having more education motivated farmers to switch to organic agriculture [5,27]. There was a significantly higher percent of conventional farmers who were men and who reported having stress symptoms in the past 2–4 weeks. However, almost twice as many organic farmers had second jobs compared to conventional farmers, even though they both reported similar hours per week of farming work. The percent of conventional farmers who smoked cigarettes, drank alcoholic beverages, and sprayed insecticides at home was significantly higher than the corresponding values for organic farmers.

To account for potential risk factors that could be associated with thyroid levels, we examined these potential associations in univariate models, then controlled for sex, smoking, drinking alcohol, insecticide use at home, triglyceride levels, and stress in our final models. We still found that conventional farmers had significantly higher TSH, FT3, T3, and T4 levels compared to organic farmers. These findings align with Piccoli et al. (2016) [13], who reported increased TSH levels in the high pesticide use season in Brazil. Farokhi and Taravati (2014) [28] reported that TSH levels were significantly increased but T3 levels were significantly decreased in sprayers using organophosphate and organochlorine insecticides, compared to those in the control group. Among the female spouses in the U.S. Agricultural Health Study, Goldner et al. (2010) [26] reported increased odds of hypothyroidism among those reporting ever using organochlorine insecticides or fungicides, but not with those reporting ever having used herbicides, fumigants, organophosphates, pyrethroids, or carbamates. In our study, farmers used a variety of pesticides over the growing season, sometimes in combination. These included mixtures of insecticides, herbicides, or fungicides. Mechanisms for thyroid disruption by pesticides may include interference at the hypothalamic pituitary thyroid (HPT) axis, inhibition of iodine intake by the thyroid gland, increased excretion of thyroid hormones, decreased cellular uptake of thyroid hormones, and up or down regulated expression of thyroid hormone regulated genes [25,29,30].

To understand which pesticides might be driving the increased risk of conventional farmers, we used models of the thyroid hormone levels that included the moles sprayed of the active ingredients of different pesticides (insecticides, herbicides, fungicides). We found that small but significant increases in TSH were associated with an increase in the moles of herbicides applied in the past year. Piccoli et al. (2016) [13] reported that in men, life time years of herbicide use was associated with increased TSH and decreased FT4, and also that lifetime use of fungicides and the insecticide dithiocarbamate was significantly associated with increasing TSH and decreasing FT4 in men. We did not see a significant association between the moles of fungicide or insecticide used in the past year and thyroid hormone levels. However, Lacasana et al. (2010) [9] reported a significant increase in TSH and T4 with an increase in an organophosphate insecticide metabolite (dimethylphosphate) in urine. In studies of fish, the insecticide cypermethrin was found to increase serum TSH and decrease T3 and T4 hormones [31].

We tried to identify which individual herbicide was most important in altering thyroid levels, and found that as the moles sprayed increased, specific herbicides significantly increased levels of different thyroid hormones: paraquat (TSH, FT3 and T3), atrazine (TSH, FT4 and T3), glyphosate (T4), diuron (TSH), acetochlor (FT4), and “other” herbicides, which included alachlor, propanil, and butachlor (FT4 and T3). Goldner et al. (2010) [26] observed a significant association between ever using paraquat and self-reported hypothyroidism among women in the U.S. Agricultural Health Study (AHS), but they did not find an association between self-reported use of paraquat and hypothyroidism in male private pesticide applicators [29]. Shrestha et al. (2018) [32] did not observe an association of incident cases of hypothyroidism in relation to paraquat use for either male or female pesticide applicators in an extended follow-up of the AHS cohort. Tsatsakis et al. (1996) [33] reported that postmortem analysis of humans with paraquat poisoning revealed detectable amounts of paraquat in the thyroid gland. They suggested that the thyroid could be susceptible to the effects of paraquat. We found increasing use of glyphosate (moles sprayed) significantly increased T4 compared to farmers not using it. Shrestha et al. (2018) reported an increasing risk of self-reported incident hypothyroidism among those reporting ever using glyphosate. However, Goldners et al. (2010) [26] and Lerro et al. (2018) [34] did not observe a significant association between glyphosate users and a thyroid effect. 

We did not find an association with moles sprayed of 2,4-D and thyroid hormone levels. However, Shrestha et al. (2018) [32] found an increase in self-reported incident hypothyroidism in pesticide applicators who reported ever using 2,4-D. Goldner et al. (2010) [26] did not find a significant association between reports of ever using 2,4-D and hypothyroidism among female spouses of applicator/farmers, but they found a significant association between reports of ever using 2,4-D and hypothyroidism in male private pesticide applicators. We found that increased moles sprayed of atrazine significantly increased TSH and T3 but decreased FT4. Goldner et al. (2010) [26] and Shrestha et al. (2018) did not observe significant associations between atrazine use and thyroid disease. For other herbicides including alachlor, propanil, and butachlor, we found that increasing moles sprayed increased FT4 and T3. Goldner et al. (2010) [26] did not observe an association of alachlor with self-reports of hypothyroid/hyperthyroid in female spouses of AHS applicators/farmers, but Goldner et al. (2013) [29] observed a significant association between self-reported ever use of alachlor and hyperthyroidism.

This study is part of a larger longitudinal study of metabolic disorders among Thai conventional and organic farmers. The findings of this cross-sectional study, which forms the baseline of the longitudinal data collection, suggest that pesticides could alter thyroid hormone status. We postulate that, in turn, altered thyroid hormone status could alter lipid metabolism, resulting in an increase in the risk factors for metabolic disorders (obesity, high triglycerides, low HDL, high blood pressure, high glucose, etc.).

In this study, we found that, in addition to increased thyroid hormone levels, conventional farmers were significantly more likely to have abnormal total cholesterol, HDL, LDL, BMI, and non-significantly higher triglyceride levels. In previous work, we found that even after controlling for sex, age, smoking, drinking alcohol, eating fruits and vegetables less than half a kilogram 5–7 days/week, heavy exercise, and history of pesticide use and insecticide use at home, conventional farmers had a significantly higher risk of abnormal total cholesterol, HDL, LDL, triglycerides, % body fat, and waist circumference than organic farmers [35]. These findings suggest that alteration of the endocrine system and thyroid hormone levels by pesticide exposure may be on the causal pathway to metabolic disorders.

This study is the first in Thailand to examine the relationship between pesticide exposure and endocrine disruption of thyroid function. It included the detailed collection of current pesticide use, allowing us to estimate the moles of active ingredients applied over a year. A limitation of this study is that it represents a single cross-sectional measurement of thyroid hormone levels. Under- or over- estimates of some parameters may have occurred in the final results. We are currently collecting longitudinal data (repeated measures) on this cohort to examine trends in these hormone levels, among other outcomes. Although we controlled for gender in our models, we should still point out that we had more females in the organic farming group, and on average, the organic farmers were 4 years older. The incidence of thyroid disease in women is 5–20 times higher than in men, and it increases with age [36]. As women approach menopause, estrogen levels decrease, which will affect thyroid hormone levels as well, regardless of the impact of pesticides exposure. This may be a partial explanation for the significantly higher proportion of hypothyroid subjects among the organic farmers. Unfortunately, we did not collect data on the menopausal status of this cohort. Another limitation is that the diary collection may have been incomplete, since we have no way to verify the use by the farmers. In addition, most of our currently organic farmer control group had previously used chemical pesticides for an average of 16 years (range 0–40), compared to the conventional farmers who have used pesticides for an average of 27 years (range 4–51) [35]. Another limitation is that conventional farmers use many types of pesticides including insecticides, herbicides, and fungicides, so attributing risk can be difficult, as some of the observed associations may be the result of interactions between pesticides, acting as either agonist or antagonists to thyroid hormone action [13].

## 5. Conclusions

This study examined the effect of pesticides on the thyroid hormone levels (TSH, T3, T4, FT3, and FT4) of conventional farmers compared to organic farmers. The TSH, FT3, T3, and T4 of conventional farmers were 1.6, 1.2, 1.3, and 1.1 times higher than those of organic farmers, respectively, after adjusting for confounding factors. The most common herbicide used among conventional farmers was glyphosate (69.2%), followed by paraquat, 2,4-D, acetochlor, diuron, amethrin, and atrazine. Specific herbicides had a significant relationship between the moles of active ingredient applied and change in thyroid hormone levels, after covariate adjustment including paraquat (TSH, FT3 and T3); atrazine (TSH, FT4 and T3); glyphosate (T4); diuron (TSH); Acetochlor (FT4) and the “other” herbicides, which included alachlor, propanil, and butachlor (FT4 and T3). Exposure to pesticides could impact the development of metabolic diseases by altering the endocrine system (thyroid hormone levels through the hypothalamic pituitary thyroid (HPT) axis).

## Figures and Tables

**Table 1:** Characteristic and risk factors of conventional farmers (*n* = 195) and organic farmers (*n* = 222).

Variables	Conventional Farmers, *n* (%)	Organic Farmers, *n* (%)	*p*-Value
Age (Year)			
Min-max	18–69	28–79	
Mean(SD)	50.0 (11.3)	53.1 (10.4)	0.004 ^§^
Sex			
Male	144 (73.8)	114 (51.4)	<0.001 ^†^
Female	51 (26.2)	108 (48.6)	
Educational Level			
Below elementary	11 (5.7)	4 (1.8)	0.109 ^†^
Elementary	109 (56.2)	119 (54.6)	
High school	69 (35.6)	84 (38.5)	
Bachelor or higher	5 (2.6)	11 (5)	
Marital Status			
Single	21 (11.1)	13 (6)	0.044 ^†^
Married	161 (84.7)	183 (85.1)	
Widowed/divorced	8 (4.2)	19 (8.8)	
Agricultural Work Time (Hr/Week)			
Mean(SD)	26.2 (13.8)	28.7 (17.3)	0.130 ^§^
Have Second Job			
Yes	50 (25.9)	124 (56.6)	<0.001 ^†^
No	143 (74.1)	95 (43.4)	
Second Job Work Time (Hr/Week)			
Mean(SD)	25.2 (13.7)	25.4 (17.4)	0.933 ^§^
Alcohol intake			
Current drinker	122 (62.6)	90 (40.9)	<0.001 ^†^
Non drinker	73 (37.4)	130 (59.1)	
Smoking			
Current smoker	51 (26.3)	35 (15.8)	0.009 ^†^
Non smoker	143 (73.7)	186 (84.2)	
Any Stress Symptom in Past 2–4 Weeks			
Yes	108 (55.4)	100 (45.5)	0.043 ^†^
Almost Never	87 (44.6)	120 (54.5)	
Insecticide Use in Home in the Past Year			
Yes	176 (90.3)	32 (14.5)	<0.001 ^†^
No	19 (9.7)	189 (85.5)	
Total Cholesterol (mg/dL)			
Normal (≤200)	50 (23.5)	123 (55.4)	<0.001 ^†^
Abnormal (>200)	163 (76.5)	99 (44.6)	
Triglyceride (mg/dL)			
Normal (150)	111 (57.2)	143 (64.4)	0.133 ^†^
Abnormal (>150)	83 (42.8)	79 (35.6)	
HDL (mg/dL)			
Normal (≤60)	52 (26.8)	15 (6.8)	<0.001 ^†^
Abnormal (<60)	142 (73.2)	207 (93.2)	
LDL(mg/dL)			
Normal (≤100)	21 (10.8)	63 (28.4)	<0.001 ^†^
Abnormal (>100)	173 (89.2)	159 (71.6)	
BMI (kg/m^2^)			
Normal (<18.49-24.99)	114 (58.8)	165 (74.3)	0.001 ^†^
Abnormal (≥25.00)	80 (41.2)	57 (25.7)	
Blood Pressure (mmHg)			
Normal (<140 and <90)	113 (61.1)	142 (68.9)	0.104 ^†^
Abnormal (≥140 and ≥90)	72 (38.9)	64 (31.1)	
Blood Glucose (mg/dL)			
Normal (≤125)	169 (86.7)	202 (91)	0.160 ^†^
Abnormal (>126)	26 (13.3)	20 (9)	
TSH (µIU/ml)			
Hypo (<0.34)	6 (3.1%)	14 (6.4)	0.259 ^‡^
Normal (0.34–5.60)	186 (95.9)	205 (93.2)	
Hyper (>5.60)	2 (1)	1 (0.5)	
FT3(ng/dL)			
Hypo (<0.23)	0	20 (9)	<0.001 ^‡^
Normal (0.23–0.49)	192 (99)	198 (89.2)	
Hyper (>0.49)	2 (1)	4 (1.8)	
FT4 (ng/dL)			
Hypo (<0.59)	3 (1.6)	3 (1.4)	1.000 ^‡^
Normal (0.59–1.54)	189 (97.9)	218 (98.2)	
Hyper (>1.54)	1 (0.5)	1 (0.5)	
T4 (µg/dL)			
Hypo (<6.09)	9 (4.6)	33 (14.9)	0.001 ^‡^
Normal (6.09–12.23)	181 (93.3)	179 (80.6)	
Hyper (>12.23)	4 (2.1)	10 (4.5)	
T3 (µg/dL)			
Hypo (<0.87)	29 (14.9)	91 (41)	<0.001 ^‡^
Normal (0.87–1.78)	161 (83)	127 (57.2)	
Hyper (>1.78)	4 (2.1)	4 (1.8)	

*p*-value from Chi square test ^†^, independent *t* test ^§^, Fischer exact test ^‡^.

**Table 2:** Thyroid hormone for conventional farmers (*n* = 195) and organic farmers (*n* = 222).

Thyroid Hormone	Conventional Farmers	Organic Farmers	Ratio of Thyroid Hormone ^a^	*p*-Value
TSH (µIU/mL)				
Geometric mean	1.26	0.89	1.42	<0.001
Min-max	0.06–7.07	0.02–8.51		
FT3 (ng/dL)				
Geometric mean	0.34	0.31	1.10	<0.001
Min-max	0.25–0.64	0.16–0.90		
FT4 (ng/dL)				
Geometric mean	0.82	0.82	1.00	0.810
Min-max	0.55–1.63	0.52–1.21		
T3 (µg/dL)				
Geometric mean	1.03	0.87	1.18	<0.001
Min-max	0.55–1.92	0.30–2.50		
T4 (µg/dL)				
Geometric mean	8.37	8.07	1.04	0.128
Min-max	4.10–14.05	3.05–16.31		

^a^ Ratio of geometric mean of thyroid hormone between conventional and organic farmers. *p* from independent *t* test of the log(e) concentrations.

**Table 3:** Comparison of log(e) thyroid hormone levels for conventional farmers versus organic farmers using generalized linear model.

Thyroid Hormone	Exp^β^ (95% CI) ^a^ For Conventional vs. Organic Farmers
TSH	1.609 (1.302–1.988)
FT3	1.156 (1.098–1.218)
FT4	1.005 (0.958–1.055)
T3	1.340 (1.240–1.447)
T4	1.133 (1.053–1.218)

^a^ models adjusted for sex, current smoking, current alcohol use, insecticide use at home in the past year, triglyceride levels and any stress symptoms in the past 2–4 weeks.

**Table 4:** Generalized linear regression models of thyroid hormone levels log(e) predicted by moles of different types of pesticides used in the past year for both conventional and organic farmers. Each row is a separate model.

Thyroid Hormone	Moles of Active Ingredients in Category of Pesticide Used in Past Year	Exp^β^ (95% CI) ^a^
TSH	Herbicide	1.002 (1.001–1.003)
	Insecticide	0.994 (0.967–1.022)
	Fungicide	1.054 (0.996–1.116)
FT3	Herbicide	1.000 (1.000–1.001)
	Insecticide	0.998 (0.992–1.004)
	Fungicide	1.003 (0.989–1.016)
FT4	Herbicide	0.999 (0.999–1.000)
	Insecticide	1.001 (0.995–1.006)
	Fungicide	1.008 (0.987–1.030)
T3	Herbicide	1.001 (1.000–1.001)
	Insecticide	1.000 (0.989–1.012)
	Fungicide	1.012 (0.986–1.039)
T4	Herbicide	1.000 (1.000–1.001)
	Insecticide	1.005 (0.999–1.011)
	Fungicide	0.984 (0.968–1.002)

^a^ Adjusted for sex, current smoking, current alcohol use, insecticide use at home in the past year, triglyceride levels and any stress symptoms in the past 2–4 weeks.

**Table 5:** Herbicide use patterns among conventional farmers (*n* = 195).

Herbicide	% of Conventional Farmers Using Pesticide	Geometric Mean of Moles Applied in Past Year (Min-Max)
Glyphosate	135 (69.2)	2.19 (0–23.98)
Paraquat	129 (66.2)	2.86 (0–55.90)
2,4-D	120 (61.5)	6.88 (0–279.54)
Diuron	56 (28.7)	26.28 (0–140.72)
Acetochlor	64 (32.8)	14.74 (0–87.67)
Ametrin	49 (25.1)	26.51 (0–122.29)
Atrazine	7 (3.6)	14.33 (0–38.52)
Other	60 (30.8)	1.30 (0–41.17)

Other = alachlor, propanil, and butachlor.

**Table 6:** Generalized linear regression models of log(e) thyroid hormone levels predicted by the moles of individual herbicide active ingredients sprayed in the past year for both conventional and organic farmers. Each row is a separate model.

Thyroid Hormone	Mole of Herbicide Sprayed/Year	Exp^β^ (95% CI) ^a^
TSH	Glyphosate	0.992 (0.957–1.027)
	Paraquat	1.024 (1.011–1.037)
	2,4D	0.999 (0.997–1.002)
	Diuron	1.007 (1.002–1.011)
	Acetochlor	0.994 (0.988–1.001)
	Ametrin	0.995 (0.990–1.001)
	Atrazine	1.022 (1.009–1.035)
	Other ^b^	1.007 (0.991–1.022)
FT3	Glyphosate	1.002 (0.998–1.007)
	Paraquat	1.004 (1.002–1.006)
	2,4D	1.000 (0.999–1.000)
	Diuron	0.999 (0.998–1.001)
	Acetochlor	1.000 (0.999–1.002)
	Ametrin	1.000 (0.999–1.002)
	Atrazine	1.002 (1.000–1.005)
	Other ^b^	1.004 (1.000–1.009)
FT4	Glyphosate	0.999 (0.993–1.005)
	Paraquat	0.997 (0.994–1.001)
	2,4D	1.000 (0.999–1.001)
	Diuron	0.998 (0.997–1.000)
	Acetochlor	1.002 (1.001–1.004)
	Ametrin	0.999 (0.998–1.001)
	Atrazine	0.995 (0.992–0.998)
	Other ^b^	1.010 (1.004–1.016)
T3	Glyphosate	1.006 (0.999–1.012)
	Paraquat	1.005 (1.002–1.008)
	2,4D	0.999 (0.998–1.000)
	Diuron	1.000 (0.999–1.002)
	Acetochlor	1.000 (0.998–1.003)
	Ametrin	1.001 (1.000–1.003)
	Atrazine	1.008 (1.003–1.014)
	Other ^b^	1.009 (1.002–1.017)
T4	Glyphosate	1.007 (1.001–1.014)
	Paraquat	1.001 (0.998–1.005)
	2,4D	0.999 (0.998–1.000)
	Diuron	1.000 (0.999–1.004)
	Acetochlor	1.002 (1.000–1.005)
	Ametrin	1.002 (1.000–1.004)
	Atrazine	1.000 (0.995–1.006)
	Other ^b^	1.004 (0.998–1.009)

^a^ Adjusted for sex, current smoking, current alcohol use, insecticide use at home in the past year, triglyceride levels and any stress symptoms in the past 2–4 weeks. ^b^ Other = alachlor, propanil, and butachlor.
